# Obesity in young children and its relationship with diagnosis of asthma, vitamin D deficiency, iron deficiency, specific allergies and flat-footedness: A systematic review and meta-analysis

**DOI:** 10.1111/obr.13129

**Published:** 2020-08-18

**Authors:** Stephen Malden, Jenny Gillespie, Adrienne Hughes, Ann-Marie Gibson, Abdulaziz Farooq, Anne Martin, Carolyn Summerbell, John J. Reilly

**Affiliations:** 1 Physical activity for Health group, School of Psychological Sciences and Health, University of Strathclyde, Glasgow, UK; 2 Aspetar Orthopaedic and Sports Medicine Hospital, Athlete Health and Performance Research, Doha, Qatar; 3 MRC/CSO Social and Public Health Sciences Unit, University of Glasgow, Glasgow, UK; 4 Department of Sport and Exercise Sciences, Durham University, Durham, UK; 5 Centre for Medical Informatics, the Usher Institute, University of Edinburgh, Edinburgh, UK

**Keywords:** associations, childhood obesity, co-morbidities, meta-analysis

## Abstract

There is evidence that a number of medical conditions and co-morbidities are associated with obesity in young children. This review explored whether there is evidence of associations with other conditions or co-morbidities. Observational studies of young children (mean age < 10 years) were identified using electronic searches of five databases (MEDLINE, Embase, CINAHL, AMED and SPORTDiscus). Of 27 028 studies screened, 41 (comprising 44 comparisons) met the inclusion criteria. These studies provided data on five distinct diseases/conditions: asthma (*n* = 16), vitamin D deficiency (*n* = 10), iron deficiency (*n* = 10), allergies (*n* = 4) and flat-footedness (*n* = 4). Thirty-two studies were appropriate for meta-analysis using random-effects models, and revealed obesity was significantly associated with having asthma (OR 1.5, 95% CI 1.3–1.7), vitamin D deficiency (OR 1.9, 95% CI 1.4–2.5) and iron deficiency (OR 2.1, 95% CI 1.4–3.2). Heterogeneity (*I* ^2^) ranged from 57% to 61%. Narrative synthesis was conducted for all studies. There was no evidence of a consistent association between obesity in young children and eczema, dermatitis or rhinitis due to the low number of studies. However, there was an association with flat-footedness. These results have implications for health policy and practice and families. Further research leading to a greater understanding of the associations identified in this review is suggested.

## 1 Introduction

While having obesity in childhood is a known predictor of numerous health conditions in adulthood, ^1,2^ a growing evidence base has demonstrated the adverse health effects obesity has during childhood and adolescence. Specifically, an abundance of research has demonstrated the link between having childhood obesity and cardio-metabolic disease markers such as high cholesterol, hypertension and abnormal glucose tolerance, with children with obesity at a threefold increased risk of hypertension than children without obesity for example.^3–5^ A recent systematic review of observational studies and randomized trials demonstrated that 5- to 15-year-old children with obesity had 7.49 mmHg higher systolic blood pressure, 0.15 mmol L^−1^ higher total cholesterol, 0.26 mmol L^−1^ higher triglycerides and significantly higher fasting insulin and insulin resistance than children without obesity. ^6^ The latter finding further supports recent research demonstrating the association between having childhood obesity and the development of type 2 diabetes in youth.^7^

While the relationship between having childhood obesity and cardio-metabolic risk factors has been well established by published research, including several systematic reviews, the potential relationship between having obesity in childhood and other co-morbid conditions is not as clearly understood. Furthermore, a focus on these co-morbidities in younger children with obesity is limited to date. Epidemiological research has demonstrated associations between childhood obesity and increased risk of asthma, sleep apnoea, vitamin D deficiency, non-alcoholic fatty liver disease, dental caries, eye disorders, atopic disease and musculoskeletal complaints among other conditions.^8–16^ However, recent systematic reviews investigating the health impacts of having childhood obesity have not been definitive regarding these conditions. Specifically, asthma has increasingly been linked to having childhood obesity; however, a review by Pulgaron identified a number of studies in which no association between childhood obesity and asthma was reported.^17^ Another systematic review and meta-analysis of 48 epidemiological studies demonstrated a weak but significant link between asthma and overweight/obesity in children. ^18^ However, a systematic review of 10 longitudinal studies demonstrated that children who had obesity as a child were more likely to suffer from asthma either in childhood or in adolescence,^19^ a finding supported by an umbrella review of risk factors for childhood obesity.^20^

There are a number of plausible explanations for the equivocal findings observed for the relationship between having some co morbidities and childhood obesity. However, one issue that is rarely discussed sufficiently in the literature is the methodological issues that may arise from the practice of combining children with overweight and obesity during participant recruitment and subsequent analyses. Many observational studies grouped children with obesity and overweight as a combined exposure variable, instead of considering both conditions as two distinct groups. Participants with overweight and obesity would rarely be combined in adult studies of co-morbidities but are routinely combined in paediatric studies. Possible reasons for this may be that there is relatively low prevalence of children with obesity in some populations historically, in comparison with overweight; therefore, recruiting an adequately powered sample to measure the outcomes of interest may be more difficult if the inclusion criteria are limited to individuals with obesity. While overweight without obesity in childhood is associated with numerous health conditions, ^21^ the grouping of participants with overweight and obesity together without appropriate stratification or subgroup analysis may dilute the real relationship between true obesity and the outcome of interest, which in some cases is considerably more pronounced than the effects of having overweight alone.^6,22^ Such issues are evident from studies that have stratified by body mass index (BMI) percentile, where the risk of co-morbidity rises as BMI increases, or prevalence is higher in groups with obesity compared with groups with overweight as defined by standardized cut-offs.^11,22^

Another potential source of inconsistent findings in research on the co-morbidities of child and adolescent obesity may be the numerous different age ranges used to define ‘childhood’ among the published literature. While some studies will distinguish childhood from adolescence using either internationally recognized categories, or researcher-defined cut-offs, others will take an all-encompassing approach and group all participants together from early childhood up to late adolescence. An issue with this approach is that the physiological, behavioural and metabolic differences between a young child and an older adolescent may have a significant influence on the outcome of interest. ^23^

The aim of this current systematic review was therefore to update and synthesize the evidence base on the physical co-morbidities of childhood obesity, focusing specifically on the impact of obesity (rather than overweight) in children under 10 years old and excluding co-morbidities of childhood obesity that have been well-established by previous systematic reviews and meta-analyses.

## 2 Methods

This systematic review was prospectively registered with PROSPERO-registration number: CRD42018079387. The Preferred Reporting Items for Systematic reviews and Meta-analyses (PRISMA) checklist was used to inform the conduct and reporting of this review. The methods used were guided by two expert Cochrane reviewers (AM and CS).

### 2.1 Inclusion and exclusion criteria

Observational studies of a cross-sectional, longitudinal or case-control design were included if they reported one measure of adiposity (e.g., BMI) in childhood (WHO definition; ≤9.9 years) and measured at least one physical health outcome in either childhood or adolescence (age 0–19 years). Studies that included children older than 10 years of age were included if the mean age of the overall sample was ≤9.9 years. Therefore, the overall age range of children within the included studies was 2–19 years (a number of studies stratified by age groups). Included studies were required to contain both groups with obesity and groups without obesity as a comparison within the sample and be published in English. Studies were also required to include both children with and without the respective co-morbid conditions. Studies that only recruited children with co-morbidities were excluded.

A number of co-morbid conditions including cardiovascular disease markers, diabetes, dental carries, sleep apnoea and metabolic syndrome were not incorporated into the search strategy for this review as the relationship is either well established, or a recent good-quality systematic review has been published relating to these conditions.^24–26^ Therefore, any studies reporting exclusively on these outcomes concerning childhood obesity were excluded. However, all other potential co-morbidities of childhood obesity were deemed eligible for inclusion in the review providing they met the inclusion criteria. Studies were excluded if

- The study population had a prior health condition that would limit generalizability of the study findings (such as children born preterm or who had a disability).
- Studies that exclusively recruited children who had the outcome of interest (co-morbidity) without an unaffected comparison group (as this would not allow for the assessment of weight status on the incidence of the condition).
- All participants in the study population had the exposure (obesity) without a comparison group that did not have obesity.
- Reported exclusively non-physical conditions (i.e., mental health conditions).
- Recognized definitions of obesity were not used or reported in the study (e.g., ≥95th percentile for age and gender using national or international growth charts).
- Children with obesity and overweight were combined without stratification during analysis.
- The mean age of the sample was over 9.9 years at the time of obesity exposure.
- Anthropometry or presence of morbidities was obtained through parental or self-report (parental reporting of doctor diagnosis of condition was included).

### 2.2 Search strategy and study selection

A computerized search of five electronic bibliographic databases (MEDLINE, Embase, CINAHL, AMED within Ovid and SPORTDiscus within Ebsco database platforms) was undertaken from January 2001 to December 2016. A forward citation search was also conducted on all eligible studies up to December 2018. This allowed for the identification of relevant studies that had been published during the time that had elapsed between the original search and completion of full-text screening. A comprehensive systematic review was published in 2003 in this subject area^5^ limiting the need to search databases from their inception. The search strategy used was checked and approved by a specialist librarian in addition to an experienced systematic reviewer (AM) before being executed in two database platforms (Ovid and EBSCOhost). The search strategy consisted of three categories, namely, (i) population group, incorporating truncated terms such as ‘child*,’ ‘infan*’ and ‘adolesc*.’ (ii) Exposure, using adiposity-related terms (e.g., ‘body mass index,’ ‘obes*’ and ‘adipos*). (iii) Outcomes, where broad headings were initially used (e.g., ‘health,’ ‘comorbid* or co-morbid*’) before becoming more focused on specific health conditions that were identified as potential co-morbidities of obesity from the published literature (e.g., ‘pes planus,’ ‘asthma,’ ‘musculoskeletal diseases’). Where possible, MESH-headings were used in addition to free-text words to account for databases without the MESH function and specific conditions not covered under MESH-headings. The search was restricted to children, human subjects and primary research studies.

Identified studies were independently screened for eligibility by two reviewers (SM and JG), initially by title and abstract (a random 20% of identified papers were double-screened), before double full-text screening was conducted for all papers that were deemed eligible after title and abstract screening. In addition to the two reviewers discussing any inconsistencies in identification, where consensus could not be reached, a third reviewer (JRR) was included in the discussion to support resolution of the decision. Following full-text screening, eligible studies had their reference lists searched for potentially relevant studies, as did relevant published systematic reviews. Relevant titles were exported to an excel spreadsheet and screened using the same methods as initially applied to the articles identified through database searches.

### 2.3 Quality assessment

The quality of eligible studies was assessed using an adapted version of the Newcastle–Ottawa scale (NOS) previously used in a review by Herzog et al.^27^ The tool has been adapted to assess the quality of cross-sectional studies in addition to cohort and case–control studies. The scale assesses studies against the following criteria: (i) selection of the sample; (ii) comparability of the sample/participants; (iii) assessment of exposure and outcomes. Stars are awarded for high-quality aspects of each study against the three aforementioned criteria, with a total of nine stars available for case–control and cohort studies and eight stars available for cross-sectional designs. Studies awarded less than five stars were classed as having a high risk of bias, while an award of ≥5 stars indicated low risk of bias. The scale has been specifically designed for non-randomized studies and does not report summary scores, which have been shown to be unreliable.^28^ Two researchers independently appraised each study before discussing any disagreements. No formal overall assessment of the quality of evidence was undertaken, as this review only included observational studies, which are deemed by GRADE^29^ to be of either low to very low quality of evidence.

### 2.4 Data extraction

The following data were extracted from each study: authors, publication year, study design, study population characteristics (sample size, geographic location, age ranges, % female), method of recruitment, exposure (exposure assessment method, the definition of obesity), outcomes (outcome assessment method, the definition of outcome, number of outcomes), method of analysis, reported effect estimates (odds ratios, relative risk, proportions, prevalence and relevant confidence intervals), level of significance reported and confounders controlled for in the analysis. We also planned to collect any data on the socio-economic status (SES) of children under study, given the known association between some medical conditions and SES in adults, and this field was included in our data extraction form. Data were extracted by two researchers independently using the pre-designed form, which was piloted on a random sample of studies prior to full data extraction commencing.

### 2.5 Data synthesis and meta-analysis

Both meta-analysis and narrative synthesis were performed for this review. Due to the inconsistent nature of information reported in a number of included studies, coupled with considerable study heterogeneity, it was not possible to include all the studies in the meta-analyses. Where narrative synthesis was adopted, recommendations outlined in the Cochrane handbook of systematic reviews were followed,^30^ whereby the characteristics of each study were summarized in terms of the study design, risk of bias and study context for each outcome. This was then followed by an exploration of the similarities and differences between each studies’ findings.

Random-effects meta-analysis was conducted on three outcomes in this review: asthma, vitamin D deficiency and iron deficiency. Odds ratios and corresponding 95% confidence intervals were collated from studies reporting these results. For studies that did not report odds ratios, information was collected on the number of children with obesity versus children without obesity, in addition to the number who presented with the outcome of interest within these two exposure groups. Pooled odds ratios and 95% confidence intervals were generated using random-effects method on MetaXL meta-analysis software (Version 5.3; EpiGear International Pty Ltd). Forest plots were generated for each outcome, and funnel plots were used to visually assess publication bias. Heterogeneity and inconsistency were assessed using Cochran’s Q statistical test, with the inconsistency test (*I* ^2^ > 50%) used to indicate moderate heterogeneity.

Where a significant proportion of the studies were of high risk of bias, a sensitivity analysis was conducted to assess whether their removal from the model significantly affected the overall result. For all analyses, the level of significance was set at ≤.05.

## 3 Results

### 3.1 Description of included studies

**Figure 1:**
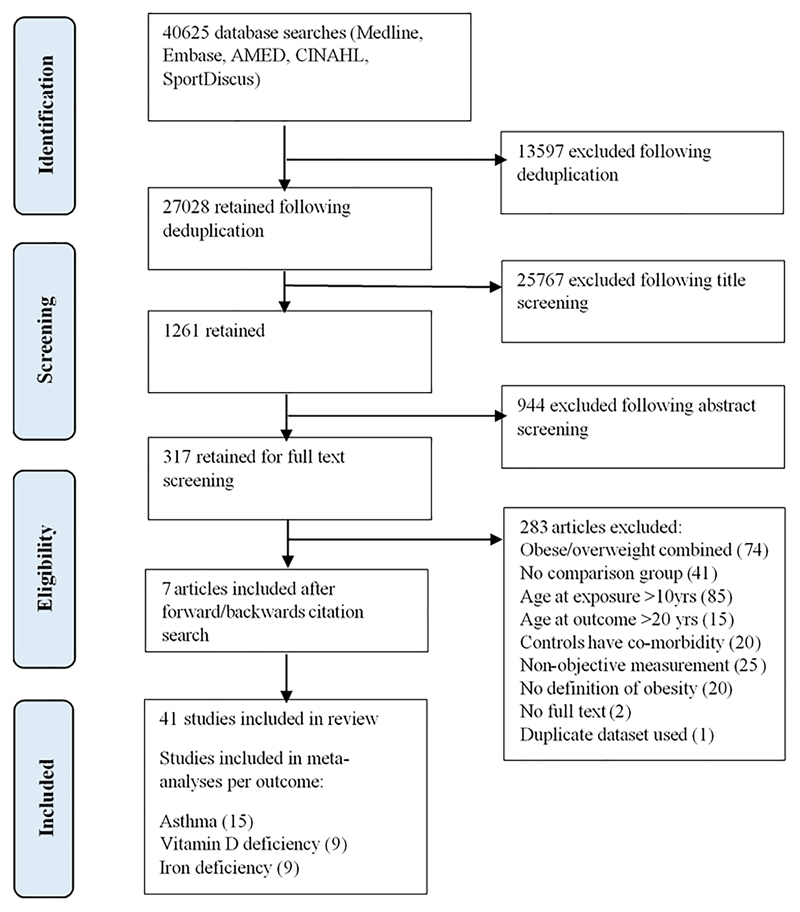
PRISMA flow diagram for study identification and inclusion

Of the 27 028 studies identified following database searches and deduplication, 41 met the inclusion criteria (Figure 1). These studies presented results investigating relationships between childhood obesity and five distinct health outcomes: asthma (*n* = 16),^31–45^ vitamin D deficiency (*n* = 10),^46–55^ iron deficiency (*n* = 10),^34,56–64^ flat-footedness/pes planus (*n* = 4)^65–68^ and allergies (*n* = 4).^34,39,69,70^ Two of the studies identified reported results for more than one of the outcomes.

Although one of the original aims of the review was to investigate obesity in children under <10 years and co-morbidity in later childhood or adolescence, we did not identify any longitudinal studies that followed young children into adolescence. Therefore, all analyses investigated the associations between having obesity and co-morbid conditions during childhood defined as under 10 years of age.

### 3.2 Study characteristics

Study characteristics are summarized in Tables 1–5. The majority of included studies were cross-sectional studies (*n* = 29), followed by case–control studies (*n* = 8). Four studies were longitudinal, three being prospective cohort studies, and one a Mendelian randomization study. Studies varied considerably by sample size, from a case–control study with 100 participants to a repeat cross-sectional study totalling 36 152 participants. All studies involved the objective measurement of anthropometry by trained practitioners. All outcomes were objectively measured using established protocols, with the exception of asthma and allergies, for which the majority of studies employed valid diagnostic survey methods to obtain confirmation via parental report of diagnosis by a health professional.

### 3.3 Study quality

Overall, cohort studies were rated as having a lower risk of bias than the case–control studies, with mean NOS scores of 7.4/9 and 6.3/9, respectively (a higher score equates to a lower risk of bias). Crosssectional studies had a mean score of 6.3 out of a possible 8 stars. In general, studies of all designs did not adequately describe or justify sample sizes or demonstrate the representativeness of the sample to the general population. Because of the stringent inclusion criteria adopted for this review, the included studies all scored highly on the NOS items pertaining to ‘assessment of exposures and outcomes.’ Six studies received a rating of below 5 stars (four cross-sectional and two case–control) and were deemed to have a high risk of bias. The NOS score and corresponding risk of bias for each study are summarized in Tables 1–5.

### 3.4 Association between childhood obesity and asthma

**Figure 2:**
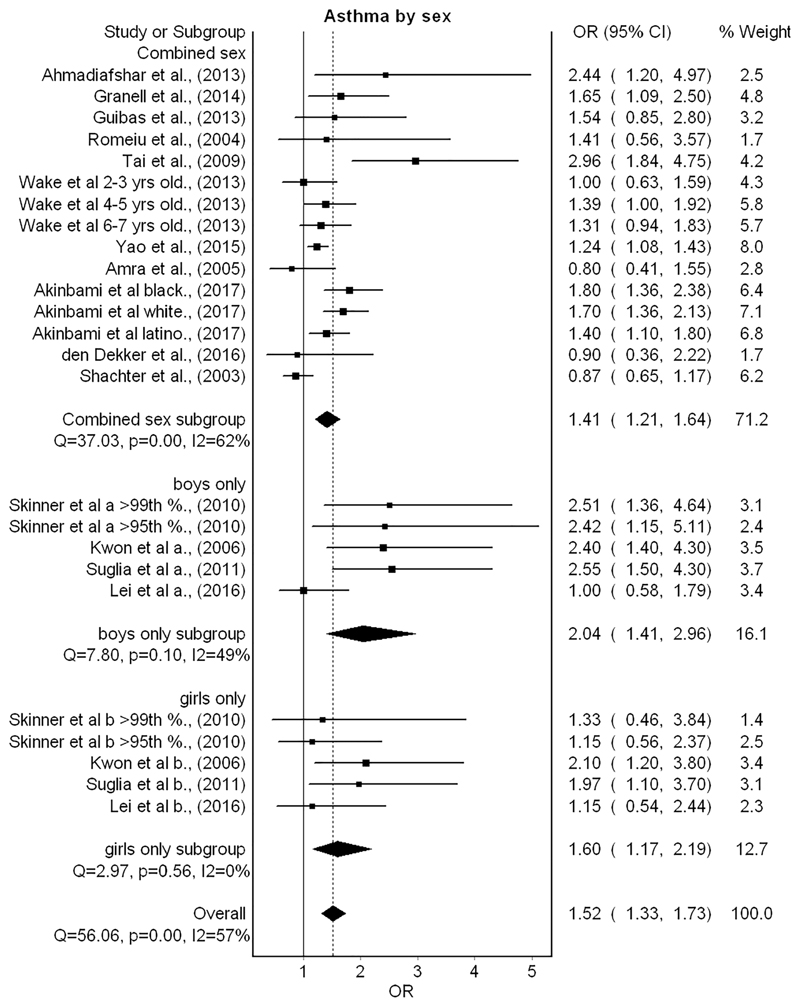
Forest plot for random effects meta-analysis of studies investigating relationships between childhood obesity and asthma (OR = odds ratio; CI = confidence interval)

**Figure 3:**
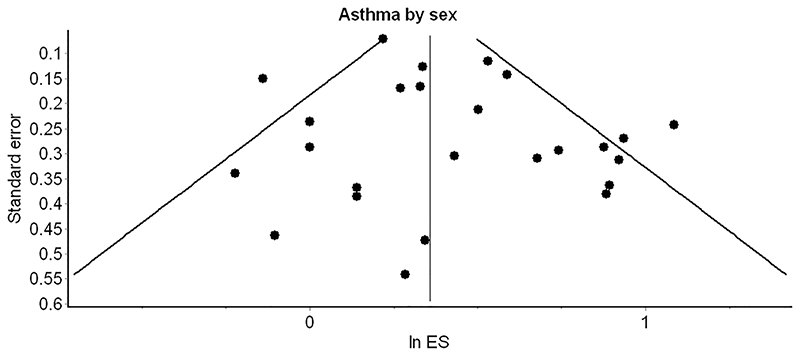
Funnel plot for studies reporting on the relationship between obesity and asthma in Meta-analysis. (ES = effect size)

Fifteen studies were grouped comparing the odds of asthma diagnosis between children with and without obesity.^31–45,71^ Six studies presented results separately for different subgroups within the study sample (i.e., by age group and ethnicity),^32,34,38,39,42,44^ and these results are presented separately in the forest plot output (Figure 2). Additionally, subgroup analysis of four studies that presented results separately for boys and girls is also included in the forest plot. The metaanalysis demonstrated that having childhood obesity significantly increased the odds of asthma diagnosis by over 50% in comparison with children without obesity (OR 1.5; 95% CI 1.3–1.7). Inconsistency was moderate (*I* ^2^ = 57%). In subgroup analysis by sex, boys showed higher odds than girls for having asthma and obesity (OR 2.0; 95% CI 1.4–2.9 and OR 1.6; 95% CI 1.2–2.2, respectively). However, this finding was not statistically significant *(p* > .05). Two of the studies were assessed as having a high risk of bias.^31,33^ However, removal of these studies from the model in a sensitivity analysis did not lead to a statistically significant change in the pooled result (OR 1.5; 95% CI 1.3–1.7). No publication bias was indicated by the funnel plot (Figure 3).

One additional study met the inclusion criteria for the review but was not appropriate for inclusion in the meta-analysis, as it was a large prospective cohort study,^71^ which presented time-to-event analysis that would be poorly interpreted by conversion to odds. The study was evaluated as having a low risk of bias and reported that having moderate obesity (≥95th centile) and extreme obesity (≥99th centile) both significantly increased the risk of asthma diagnosis.^71^

### 3.5 Association between childhood obesity and vitamin D deficiency

**Figure 4:**
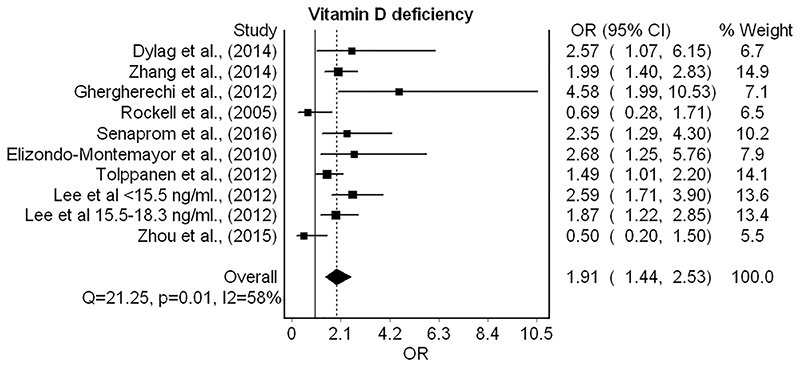
Forest plot for random effects meta-analysis of studies investigating relationships between childhood obesity and vitamin D deficiency

Nine separate studies were included in the meta-analysis of vitamin D deficiency and obesity,^46–48,50–55^ resulting in a pooled odds ratio of 1.9 (95% CI 1.4–2.5) (Figure 4). The studies showed moderate heterogeneity (*I* ^2^ = 58%). The majority of studies defined deficiency as <20 ng ml^−1^, and one study reported separate odds ratios for deficiency and severe deficiency, which have been added to the forest plot separately.^50^ Studies that reported serum levels in nmol L^−1^ were less uniform, with deficiency cut-offs ranging from <17.5 to <30 nmol L^−151,55^ (see Table 2). Two of the studies included in the meta-analysis had a high risk of bias.^46,48^ When these studies were removed during sensitivity analysis, the pooled effect size was reduced to OR 1.7 (95% CI 1.3–2.3).

One additional study^49^ reported insufficient data to allow for pooling within the meta-analysis and was judged as having a high risk of bias.^49^ This study reported significantly lower vitamin D levels in children with obesity, due to its small sample size, this study would not have significantly influenced the overall effect size in pooled analysis had it been included.

### 3.6 Association between childhood obesity and iron deficiency

**Figure 5:**
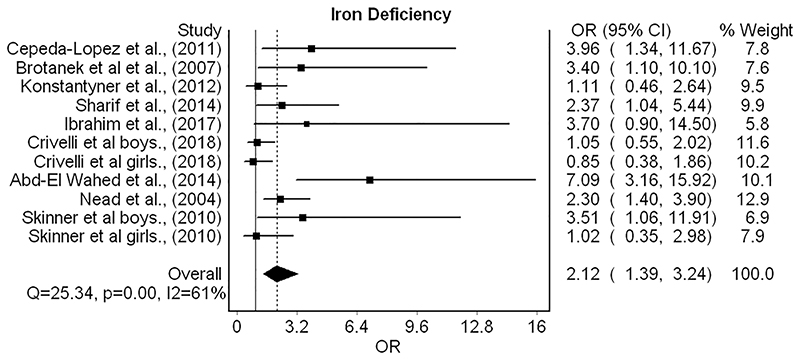
Forest plot for random effects meta-analysis of studies investigating relationships between childhood obesity and iron deficiency

Ten studies investigated the relationship between childhood obesity and iron deficiency^34,56–64^ of which nine were appropriate for meta-analysis (Figure 5). Two of these studies conducted separate analyses by sex,^34,59^ with both subgroups included in the model individually. Meta-analysis revealed that having obesity doubled the odds of iron deficiency diagnosis (OR 2.1; 95% CI 1.4–3.2). However, the removal of one study^56^ with a large effect size during sensitivity analysis reduced the association (OR 1.8; 95% CI 1.3–2.6).

One case–control study^60^ assessed as having a low risk of bias was not appropriate for meta-analysis due to insufficient reporting of data necessary for calculation of odds and found children with obesity to have significantly different markers of iron deficiency than the control group. Specifically, children with obesity had significantly lower iron, transferrin saturation and total-iron binding capacity along with higher ferritin, soluble transferrin receptors and hepcidin-25 than children of normal weight.

### 3.7 Association between childhood obesity and pes planus (flat-footedness)

Four studies investigated the relationship between childhood obesity and having flat-footedness.^65–68^ Of these, one was a longitudinal study,^65^ two were of a cross-sectional design^66,67^ and one was a case control study.^68^ All four studies were of low risk of bias and reported a statistically significant association between having flat-footedness and obesity. One study^66^ investigated both bilateral and unilateral flat-footedness but only found having obesity to significantly increase the odds of the bilateral condition (OR 1.9; 95% CI 1.2–2.9), while the remaining three studies investigated bilateral flat-footedness only. All four studies were assessed as having a low risk of bias following quality assessment (Table 4).

### 3.8 Association between childhood obesity and allergies

Four studies assessed the relationship between childhood obesity and allergic conditions.^34,39,69,70^ Two distinct conditions were investigated within the studies; all four studies assessed eczema/dermatitis, and one study also included rhinitis as an outcome.^39^ Additionally, one study reported results from the skin prick test.^70^ Three of these studies had a cross-sectional design, and one was a case–control study.^69^ All four studies had a low risk of bias (Table 5). Three of the four studies found having obesity to increase the odds of eczema/dermatitis diagnosis; however, these effects were small to moderate.^39,69,70^ One study reported no association between eczema and obesity/severe obesity.^34^ Having obesity was found to slightly increase the odds of rhinitis diagnosis in one study (OR 1.3; 95% CI 1.0–1.7) when the sample was analysed collectively. However, a differential effect by sex was reported, as the association was only evident in girls and not boys.^39^ Obesity was not found to significantly increase the odds of a positive skin prick test (OR 1.1; 95% CI 0.9–1.4).

## 4 Discussion

This systematic review and meta-analysis investigated associations between obesity in young children and multiple co-morbid conditions. Though this topic has been studied through both primary research and recent systematic reviews,^17,18,72–78^ we investigated obesity as a distinct condition from overweight. This is in contrast to similar systematic reviews in the subject area, which have included studies that combine individuals with overweight or obesity, or do not stratify by weight status in the analysis.^17–19,73,78^ We also used more stringent inclusion criteria to define childhood as children under 10 years, potentially offsetting the physiological, cultural and behavioural effects that later childhood/adolescence can have on obesity co-morbidities.^79,80^

The results of our review offer a number of important findings. Firstly, the meta-analysis of childhood obesity and asthma diagnosis appears to support previous results from systematic reviews in this area,^18,77,78^ while also further distinguishing the effects of having obesity considered explicitly from overweight. Chen et al.^77^ reported a significantly higher risk of incident asthma among children and adolescents with obesity compared with children without obesity (relative risk 2.02; 95% CI 1.16–3.50), while a narrative synthesis by Papoutsakis et al.^18^ concluded that there was a clear relationship between childhood obesity and asthma incidence. Our finding that having obesity at a young age increased the odds of asthma by over 50% indicates that there is a possible relationship between the two conditions. However, we did not identify any longitudinal studies that were appropriate for meta-analysis based on our inclusion criteria, meaning the cross-sectional data that our findings are based on cannot offer any indication of a causal link between obesity and asthma. One cohort study included in our narrative synthesis found that not only did having a higher BMI predispose children to subsequent asthma development, but children with both asthma and obesity or overweight were also more likely to develop a severe asthma phenotype than healthy weight children with asthma.^71^ A meta-analysis of six longitudinal studies also found that asthma risk increased by 35% among children with obesity/overweight.^78^ Conversely, Chen et al. reported that asthma in fact preceded the onset of obesity even after controlling for glucocorticosteroid usage, with children with asthma having 51% higher risk of developing obesity at follow-up than children without asthma.^81^

There may be legitimate physiological and behavioural explanations for both directions of the relationship. Firstly, physiological consequences of obesity such as reduced lung and tidal volume, low-grade systemic inflammation and changes in adipose-derived hormones likely promote the onset of asthma.^82^ Conversely, children with normal weight and asthma may be at a higher risk of developing overweight and obesity due to the observed tendency for children with asthma to avoid moderate-vigorous physical activity,^83,84^ an important protective factor against excess weight gain.^85^ Additionally, asthma medications such as glucocorticosteroids are theorized to promote weight gain through increased lipid metabolism and storage.^81^

Of the four identified studies relating to musculoskeletal disorders included in our review, all related to flat-footedness, and all found having obesity to significantly increase risk. Other musculoskeletal disorders have been studied in relation to childhood obesity; however, these did not meet our inclusion criteria. A recent review by Paulis and colleagues found musculoskeletal pain to be related to childhood overweight and obesity,^73^ supporting the findings of our review that obesity may have structural/biomechanical consequences. Potential physiological explanations for this are expressed in the literature, with excess fat deposits on feet or excess load-bearing due to excess weight causing arches to collapse in children with obesity.^68^

This review identified vitamin D deficiency as a condition that is associated with obesity in young children, a finding that has only previously been investigated in a small number of systematic reviews.^17,74,75^ Periera-Santos and colleagues found that obesity in children and adolescents increased the prevalence of vitamin D deficiency by 37% in a meta-analysis of eight studies.^74^ Another meta-analysis reported a pooled odds ratio of 3.43 (95% CI 2.33–5.06).^75^ Our finding from the present review that having obesity increases the odds of vitamin D deficiency further supports these findings. However, as with asthma, the studies that met the inclusion criteria for this review were all cross-sectional in nature, and therefore, a causal relationship could not be confirmed. Physiological mechanisms of vitamin D deficiency as a consequence of obesity have been discussed in the literature^86^; however, the nature of the relationship is still poorly understood. A longitudinal study of Colombian 5–12 year olds found vitamin D deficient participants had a 0.1/year greater change in BMI than vitamin D sufficient children,^9^ indicating that the physiological effects of obesity such as impaired hydroxylation may contribute to lower vitamin D levels in children with obesity.^87^ It is also important to consider that vitamin D deficiency has been shown to increase asthma severity, which may indicate each condition may mediate any relationship with obesity.^88^ Despite this, few studies included in this review controlled for this potential confounding (Tables 1 and 2). Furthermore, it is theorized that having obesity may impair the bioavailability of vitamin D for bloodstream absorption, as it is fat-soluble, and instead stored in adipose tissue reservoirs,^89^ further highlighting the complexity of the relationship between the two conditions.

This review also found that childhood obesity increased the odds of having iron deficiency, which to our knowledge is only the second such meta-analysis to demonstrate this relationship and the first to do so exclusively in children aged <10 years.^76^ An important observation that applies to both vitamin D and iron deficiency is that they are both nutritional deficiencies. It could therefore be that causation may be related to diet quality, as children with obesity have been shown to have poorer nutritional intake (lower nutrient density, consuming less iron-rich foods) than children with normal weight in epidemiological studies.^90^ A small number of studies included in our review controlled for diet in their analysis,^47,49,58^ still finding the conditions to be associated with obesity. However, the majority of studies concerning vitamin D and iron status did not include diet/nutrient intake as a covariate, which may have confounded the results obtained for these studies. In the case of vitamin D, this can be extended to include time spent outdoors (or physical activity as a proxy measure of ultraviolet light exposure), as it has been demonstrated that children with obesity spend less time in outdoor play and longer periods sedentary indoors.^91^ With vitamin D levels mediated by sunlight exposure,^47^ this could potentially explain the differences observed in children with obesity from a behavioural perspective. Therefore, the results of a number of included studies that did not control for these covariates should be interpreted with caution.^46,48,50–53,55^ In the case of iron deficiency, while mechanisms explaining effects of obesity on the condition are not fully understood, individuals with obesity have both increased iron requirements (secondary to increased blood volume) and reduced iron absorption (secondary to increased inflammation).^92^ Interventions to reduce weight status in children with vitamin D and iron deficiency would therefore enhance understanding of the causal effects of having obesity in these conditions.

While the inclusion criteria adopted for this review are a strength of this study, there are a number of limitations that should be considered when interpreting the findings. We decided against conducting an overall assessment of the quality of evidence for each outcome by using GRADE assessment criteria.^29^ Despite this, an alternative assessment of some key quality indicators adds further context to the strength of the evidence presented in this review. Specifically, the majority of studies were cross-sectional or case–control studies, which are more susceptible to bias than longitudinal studies of the same methodological rigour. Secondly, inconsistency was evident as the moderate–high heterogeneity observed within the meta-analyses in this review reflects the fact that a number of included studies did not adequately control for confounding factors in their analyses. It is therefore possible that the results of this review may have been affected by residual confounding and should be interpreted with caution. Definitive causal effects of childhood obesity on the co-morbid conditions identified in this review have still to be established, but plausible mechanisms have been identified as discussed above. It would therefore be beneficial for behavioural and environmental obesity treatment interventions to include measurement of morbidity as an outcome in evaluations, to determine if reductions in weight status are also accompanied by improvements in disease symptoms/presentation.

This systematic review and meta-analysis identified a number of co-morbidities of childhood obesity that were not well established previously. Evidence of an association between childhood obesity and diagnosis of asthma, vitamin D deficiency, flat-footedness and allergies is reported, in addition to the novel finding that iron deficiency is a potential co-morbidity of childhood obesity. Additionally, it appears that a better understanding of any important inequalities (by SES) in the relationship between obesity and health conditions in young children is needed to help support policy and practice with regard to obesity prevention in children.^93^ Healthcare professionals may find our results helpful when treating pediatric patients with obesity, in terms of additional assessment and consideration for the co-morbid conditions identified and investigated in this review. The potential for obesity to cause harm as early as childhood is apparent, and efforts to prevent obesity in the early years could, in turn, alleviate the health burden of conditions associated with having excess weight in childhood.

## Figures and Tables

**Table 1:** Stud ies reporting on the relationship between obesity and asthma (*n* = 16)

Author and publication year	Study design	Country	Sample size (*n*)	Age range or mean age	Obesity definition	Outcome definition	Outcome identification	Covariates	Results	Study quality (based on NOS score)
Ahmadiafshar et al.	Case control	Iran	400	6–15 years	CDC growth charts	Report of doctor diagnosis	Asthma—history, clinical findings and pulmonary function test	-	Obesity increased odds of asthma: (OR 2.44; 95% CI 1.2–4.97)	High risk of bias (4/9)
Black et al.	Prospective cohort	United States	14 987	6–10 years	CDC growth charts: moderately obese (>95th percentile OR BMI ≥ 30) and extremely obese (≥99th percentile OR BMI ≥ 35)	Asthma Index ICD-9 code 493	Parental questionnaire	Sex, race/ethnicity, insurance payer	Obesity increased the risk of asthma: moderate obese: overall (HR 1.32 95% CI 1.26–1.39), girls HR 1.36 95% CI 1.27–1.46), boys (HR 1.28 95% CI 1.21–1.36) Extreme obese: overall (HR 1.49 95% CI 1.41–1.5), girls (HR 1.56 95% CI 1.43–1.71), boys (HR 1.43 95% CI 1.33–1.5)	Low risk of bias (9/9)
Skinner et al.	Cross-sectional	United States	2792	3–5 years	≥95th percentile obese; ≥99th percentile very obese	Report of doctor diagnosis of asthma	Parental questionnaire/interview	Age, race/ethnicity, income, insurance status	Obese or very obese status increased odds of asthma in boys but not in girls: very obese: boys (OR 2.51 95% CI 1.36–4.64; girls OR 1.33 95% CI 0.46–3.84). Obese: boys (OR 2.42 95% CI 1.15–5.11; girls OR 1.15 95% CI 0.56–2.37)	Low risk of bias (8/8)
Granell et al.	Mendillian randomization	United Kingdom	4835 (2376 girls)	7 and 9 years	≥95th percentile	Report of doctor diagnosis of asthma	Parental questionnaire	-	Obesity increased the risk of asthma at 7 years old (RR 0.21; 95% CI 0.14–0.31)	Low risk of bias (9/9)
Guibas et al.	Cross-sectional	Greece	1622 (789 girls)	2–5 years	≥95th percentile	Report of doctor diagnosis of asthma	Parental questionnaire	Prenatal smoking, gestational age, birthweight, gender, parity, breastfeeding, passive smoking at home, nationality, parental educational level	Obese status did not significantly increase odds of asthma (OR 1.54; 95% CI 0.85–280)	Low risk of bias (6/8)
Kwon et al.	Cross-sectional	United States	853 (431 girls)	7.5 years	U.K. 1990 growth charts	Report of doctor or nurse diagnosis of asthma and evidence of asthma-like symptoms or asthma-related emergency care use during the past year	Parental questionnaire	Age, race/ethnicity, nativity, household smoking exposure	Obese status increased odd of asthma in boys and girls: (boys OR 2.4; 95% CI 1.4–4.3; girls OR 2.1; 95% CI 1.2–3.8)	Low risk of bias (7/8)
Romeiu et al.	Cross-sectional	United States	3337	2–5 years	IOTF cut-offs	Report of doctor diagnosis of asthma and report of current asthma symptoms	Parental questionnaire	Wheezing, atopy, physical activity, vitamin C consumption, dietary intake, race, poverty income ratio, passive smoking, parental asthma, hay fever ever	Obese status did not significantly increase the odds of asthma (OR 1.41; 95% CI.56–3.57)	Low risk of bias (7/8)
Suglia et al.	Cross-sectional	United States	1815	3 years	CDC growth charts	Report of doctor diagnosis of asthma which had been active within the last year	Parental questionnaire	Sex, race/ethnicity, low birth weight, maternal education, parent marital status, maternal age, public assistance, daycare attendance, maternal depression, intimate partner violence, child neglect, housing quality, tobacco exposure	Obese status increased odds of asthma in boys and girls (whole sample OR 2.26; 95% CI 1.5–3.3. Boys OR 2.55; 95% CI 1.5–4.3. Girls OR 1.97; 95% CI 1.1–3.7)	Low risk of bias (7/8)
Tai et al.	Cross-sectional	Australia	1509 (737 girls)	4–5 years	CDC growth charts	Report of doctor diagnosis of asthma	Parental questionnaire	Sex	Obese status increased odds of asthma (OR 2.96; 95% CI 1.84–4.75)	Low risk of Bias (5/8)
Wake et al.	Cross-sectional	Australia	13 879	2–7 years	CDC growth charts	Report of doctor diagnosis of asthma with medication use in last 12 months	Parental questionnaire	-	Asthma prevalence: 2–3 yrs = normal weight—10.1 (0.5); obese—13.5(2.4). 4–5 yrs = normal weight—14.5 (0.6); obese—19.1 (2.5). 6–7 yrs = normal weight—15.4 (0.6); obese—19.4 (2.6)	Low risk of bias (7/8)
Yao et al.	Cross-sectional	China	12 092 (5761 girls)	8.2 years	IOTF cut-offs	Report of doctor diagnosis of asthma	Parental questionnaire	Age, sex	Obese status increased the odds of asthma (OR 1.242; 95% CI .080–1.429)	Low risk of bias (5/8)
Amra et al.	Cross-sectional	Iran	2413	7–12 years	IOTF cut-offs	Report of doctor diagnosis of asthma	Parental questionnaire	Sex, age, parental smoking, family history	Obese status was significantly associated with asthma	High risk of bias (4/8)
Akinbami et al.	Repeat cross-sectional	United States	36 152	2–19 years	Cole et al.	CDC asthma surveillance definition	Parental questionnaire	Age, sex, income status	Obese status significantly increased odds of asthma in White, Black and Mexican American children (White OR 1.7; 95% CI 1.4–2.2. Black OR 1.8; 95% CI 1.6–2.1. Mexican American OR 1.4; 95% CI 1.1–1.8)	Low risk of bias (7/8)
den Dekker et al.	Cross-sectional	Netherlands	6178	6.2 years	≥95th percentile	Global Initiative for Asthma definition	Parental questionnaire	Maternal age, pre-pregnancy BMI, educational level, history of asthma and atopy, psychological distress during pregnancy, parity, smoking during pregnancy, child’s sex, gestational age at birth, birth weight, ethnicity, breastfeeding, pet keeping, physical activity, lower respiratory tract infections, current height	Obese status did not increase the odds of asthma (OR 0.90; 95% CI 0.36–2.22)	Low risk of bias (7/8)
Lei et al.	Cross-sectional	China	3327 (1663 girls)	2–14 years	CDC growth charts	Report of doctor diagnosis of asthma	Parental questionnaire	-	Obese status did not increase the odds of asthma (overall OR 1.09; 95% CI 0.68–1.72; boys OR 1.0; 95% CI 0.58–1.79; girls OR 1.15; 95% CI 0.54–2.44)	Low risk of bias (6/8)
Shachter et al.	Cross-sectional	Australia	5993 (2976 girls)	9.8 years	IOTF cut-offs	Report of doctor diagnosis of asthma, with present symptoms	Parental questionnaire	Family history of asthma, sex, atopy status, exposure to cigarette smoke	Obese status did not increase the odds of asthma (OR 0.87; 95% CI 0.65–1.17)	Low risk of bias (7/8)

Abbreviations: BMI, body mass index; CDC, Centers for Disease Control and Prevention; IOTF, International Obesity Task Force; NOS, Newcastle–Ottawa Scale.

**Table 2:** Stud ies reporting on the relationship between obesity and vitamin D deficiency (*n* = 10)

Author and publication year	Study design	Country	Sample size (*n*)	Age range or mean age	Obesity definition	Outcome definition	Outcome identification	Covariates	Results	Study quality (based on NOS score)
Dylag et al.	Cross-sectional	Poland	100 (55 girls)	1–5 years	WHO growth reference charts	Optimal vitamin D levels: >30 ≤ 50 ng ml^−1^; suboptimal vitamin D levels: ≤30 ≥ 20 ng ml^−1^; vitamin D Deficiency: <20 ng ml^−1^	Blood test/assay	Age	Significantly lower mean difference in vitamin D concentrations: 23.6 ± 10.8 obese, 26.6 ± 9.8 non-obese.	High risk of bias (4/8)
Elizondo-Montemayor et al.	Cross-sectional	Mexico	198 (98 girls)	9 years	WHO growth reference charts	Optimal vitamin D levels: ≥30 ng ml^−1^; vitamin D insufficiency: 21–29 ng ml^−1^; vitamin D deficiency: <20 ng ml^−1^	Overnight fasting blood sample assessed using competitive immunolumi nometric direct assay	Skin phototype, physical activity, screen time, vitamin use, diet	Obese status increased the odds of vitamin D deficiency (OR 2.679; 95% CI 1.245–5.765)	Low risk of bias (6/8)
Ghergherechi et al.	Case control	Iran	109	8.9 years	>95% centile for age and gender	<20 ng dl^−1^ vitamin D deficiency; <10 ng dl^−1^ severe vitamin D deficiency	Blood test/assay	Age, sex, height	Vitamin D deficiency obese group = 76.9%; non-obese = 42.1%. Severe vitamin D deficiency obese group = 44.2%; non-obese = 17.5%	High risk of bias (4/9)
Jazar et al.	Cross-sectional	Jordan	200 (100 girls)	3.3 years	CDC growth charts	Vitamin D insufficiency, from 15 to 20 ng ml^−1^; vitamin D deficiency, ≤15 ng ml^−1^; severe vitamin D deficiency ≤ 5 ng ml^−1^	Blood test/assay	Duration of breastfeeding, duration of formula feeding, duration of outdoor physical activity, calcium intake and dietary vitamin D intake	Significantly lower mean serum vitamin D levels in obese participants compared with controls (obese serum vit D levels = 13.0 ± 2.5 v normal weight 25.4 ±0.6)	High risk of bias (4/8)
Lee et al.	Cross-sectional	South Korea	1660 (756 girls)	9 years	BMI ≥ 95th percentile for age and sex	<20 ng ml^−1^ vitamin D deficient	Blood collected after overnight fasting and 25(OH)D concentrations measured by chemiluminescent immunoassay	-	Obese status increased odds of having lower mean serum vitamin D levels	Low risk of bias (5/8)
Rockell et al.	Cross-sectional	New Zealand	1585 (784 girls)	5–14 years	IOTF cut-offs	Vitamin D deficient <17.5 nmol L^−1^; vitamin D insufficient: <37.5 nmol L^−1^	Blood sample/assay	Age, ethnicity, latitude (North vs. South Island), season (‘summer’ vs. ‘winter’ months)	Both vitamin D deficiency and insufficiency were significantly associated with obese status	Low risk of bias (7/8)
Senaprom et al.	Cross-sectional	Thailand	477 (239 girls)	7.8 years	BMI-for-age Z score (BAZ) >3 SD above the median for children aged 3–5.9 years (WHO, 2006) and as a BAZ > 2 *SD* above the median for children aged 6–13 years	Vitamin D deficiency <50 nmol l^−1^	Fasting blood sample analysed by chemiluminescence immunoassay	-	Obese status was significantly associated with vitamin D deficiency	Low risk of bias (5/8)
Tolppanen et al.	Prospective cohort	United Kingdom	7555 (3744 girls)	9.8 years	Cole et al. international BMI cut-off values	Vitamin D deficiency < 20 ng ml^−1^	Non-fasting blood samples assayed using HPLC tandem mass spectrometry	-	Odds of vitamin D deficiency in obese participants was 1.49 (95% CI 1.01–2.20)	Low risk of bias (7/9)
Zhang et al.	Cross-sectional	China	1488 (656 girls)	8.8 years	Chinese obesity task force cut-off values	Vitamin D deficiency = <20 ng ml^−1^; vitamin D insufficiency = 20–30 ng ml^−1^: vitamin D sufficiency ≥ 30 ng ml^−1^	Blood sample and liquid chromatography	Age, gender, dietary energy intake, energy expenditure	Significantly higher prevalence of vitamin D deficiency among obese participants compared with normal weight	Low risk of bias (7/8)
Zhou et al.	Cross-sectional	Australia	221 (105 girls)	1–5 years	WHO growth reference charts	Deficiency = vit D < 30 nmol L^−1^; insufficiency = Vit D ≥ 30 and <50 nmol L^−1^	Non-fasting blood sample and assay	-	No significant difference between mean serum vitamin D levels in obese and normal weight individuals	Low risk of bias (6/8)

Abbreviations: BMI, body mass index; CDC, Centers for Disease Control and Prevention; HPLC, high-performance liquid chromatography; IOTF, International Obesity Task Force; NOS, Newcastle–Ottawa Scale.

**Table 3:** Stud ies reporting on the relationship between obesity and iron deficiency (*n* = 10)

Author and publication year	Study design	Country	Sample size (*n*)	Age range or mean age	Obesity definition	Outcome definition	Outcome identification	Covariates	Results	Study quality (based on NOS score)
Abd-El Wahed et al.	Case control	Egypt	120 (62 girls)	9.25 years	CDC growth charts	The presence of two or more of the following abnormal parameters: Mean corpuscular volume (MCV) is 76 fl or less; serum TS 15% or less; Serum ferritin less than 10 mg ml^−1^	Blood sample/assay	Age, sex	Obese status increased odds of iron deficiency (OR 7.09; 95% CI 3.16–15.92)	Low risk of bias (8/8)
Brotanek et al.	Cross-sectional	United States	960 (434 girls)	1–3 years	Weight-for-length status of ≥95th percentile	Ages 1–2 years, iron deficiency <10% transferrin saturation < 10 g L^−1^ of serum ferritin and >1.42 mol L^−1^ of red blood cells erythrocyte protoporphyrin. For 3-year-old children, <12% < 10 g L^−1^, and >1.24 mol L^−1^ of red blood cells	Blood sample/assay	Race/ethnicity, interview language, preschool/day care attendance	Obese status increased odds of iron deficiency (OR 3.34; 95% CI 1.10–10.12)	Low risk of bias (8/8)
Cepeda-Lopez et al.	Cross-sectional	Mexico	1174 (49% girls)	8.17 years	WHO growth reference charts	Either (1) low serum iron (<60 ug dl^−1^) or (2) elevated TIBC (>360 ug dl^−1^)and low %TS (<20%) values	Blood sample/assay	Age, sex, region, area, caregiver education	Obese status increased the odds of iron deficiency (OR 3.96; 95% CI 1.34–11.67)	Low risk of bias (7/8)
Skinner et al.	Cross-sectional	United States	2792	3–5 years	CDC growth charts	Taking medication for anaemia and laboratory values of hemoglobin <11 g dl^−1^.16	Blood sample/assay	Age, ethnicity, income, insurance status	Obese status increased the odds of anaemia in boys but not in girls (boys OR 3.51; 95% CI 1.06–11.91; girls OR 1.02; 95% CI 0.35–2.98)	Low risk of bias (8/8)
Crivelli et al.	Cross-sectional	Tajikistan	1320 (653 girls)	2–5 years	WHO growth reference charts	WHO cut-off value for iron deficiency in children (Hb < 11 g dl^−1^)	Finger prick test using Drabkin’s reagent for Hb analysis	Age, sex, location, parental education, region	Obese status did not increase odds of iron deficiency in boys or girls (boys: OR 1.05 95% CI 0.55–2.0; girls: OR 0.85 95% CI 0.38–1.86)	Low risk of bias (7/8)
Hamza et al.	Case control	Egypt	100 (42 girls)	9.8 years	Cole et al.	Fe deficient when 2 or more Fe profile values were abnormal for age and gender: serum Fe < 20 μg dl^−1^, TICB >494 μg dl^−1^, ferritin <12 μg dl^−1^, TS < 16% (2), and sTfR > 8.3 mg L^−1^	Blood sample/assay	Age	Fe, TS and TIBC were significantly lower, while ferritin, sTfR and hepcidin-25 were significantly higher in obese children versus controls	Low risk of bias (8/9)
Ibrahim et al.	Case control	Jordan	150 (61 girls)	2.1 years	WHO growth reference charts	Internationally accepted cut-off values for biochemical iron markers: Hb (g L^−1^ = 9.5–14.5; SF (ng ml^−1^) -29–160; and SI (μg dl^−1^) - 25–115.	Blood sample/assay	Age	Odds of iron deficiency in obese group compared with normal weight was 3.7 (95% CI 0.9–14.5)	Low risk of bias (6/8)
Konstantyner et al.	Cross-sectional	Brazil	1325	1–2 years	WHO growth reference charts	Mild iron deficiency anemia: Hb < 11.0 g dl^−1^; moderate iron deficiency anemia: Hb < 9.5 g dl^−1^	High-performance liquid chromatography (HPLC) of dried blood spot samples	-	Obese status did not increase the odds of mild or moderate anaemia: mild anaemia: OR 1.11 (0.46; 2.64); moderate anemia: 2.41 (0.80; 7.30)	Low risk of bias (7/8)
Nead et al.	Cross-sectional	United States	9698	2–16 years	CDC growth charts	Iron-deficient if 2 of 3 values were abnormal for age and gender. Anemia = Hemoglobin cutoff points used to define anemia were based on the fifth percentiles for the reference groups	Blood sample/assay	Age, gender, race/ethnicity, poverty status, caretaker education	Obese status increased the odds of iron deficiency (OR 2.3; 95% CI 1.4–3.9)	Low risk of bias (6/8)
Sharif et al.	Case control	Iran	100 children (49 girls)	9.5 years	CDC growth charts	Serum iron levels less than 50 μg dl^−1^ and TIBC higher than 450 μg dl^−1^ were defined as iron deficiency	Blood sample biochemistry method and plasma ferritin by ELISA method	-	Prevalence of iron deficiency significantly higher in obese versus normal weight children (48% vs. 28%)	Low risk of bias (5/9)

Abbreviations: BMI, body mass index; CDC, Centers for Disease Control and Prevention; ELISA, enzyme-linked immunosorbent assay; IOTF, International Obesity Task Force; NOS, Newcastle–Ottawa Scale.

**Table 4:** Stud ies reporting on the relationship between obesity and musculoskeletal disorders (*n* = 4)

Author and publication year	Study design	Country	Sample size (*n*)	Age range or mean age	Obesity definition	Outcome definition	Outcome identification	Covariates	Results	Study quality (based on NOS score)
Chen 1 et al.	Prospective cohort	Taiwan	580 (283 girls)	3–5 years	Taiwanese FDA definitions of obesity for children and adolescents	Flatfoot = AB distance by CSI > 62.70%. CSI is defined as the ratio of the minimum width of the midfoot arch region (B) to the maximum width of the metatarsus region (A)	Clinician measurement using digital footprint mat	Age	Prevalence of flatfoot was significantly higher in obese children	Low risk of bias (6/9)
Chen 2 et al.	Cross-sectional	Taiwan	1598 (765 girls)	3–6 years	Taiwanese FDA definitions of obesity for children and adolescents	Clinical presentations of malformation of the medial longitudinal arch in a weight bearing position	Clinician examination of foot	Age, sex, joint laxity, W sitting	Obese status increased the odds of bilateral flatfoot, but did not increase odds of unilateral flatfoot (Bilateral OR 1.90; 95% CI 1.22–2.95; unilateral OR 1.39; 95% CI 0.80–2.41)	Low risk of bias (6/8)
Ezema et al.	Cross-sectional	Nigeria	474 (253 girls)	6–10 years	CDC growth charts	Plantar arch index value >1.15	Ink footprint test	-	Prevalence of flatfoot was significantly higher in obese children	Low risk of bias (7/8)
Riddiford-Harland et al.	Case control	Australia	150 (98 girls)	8.3 years	Cole et al.	Clinical presentation of reduced foot arch on ultrasound	Ultrasound	Age, sex	Prevalence of flatfoot was significantly higher in obese children	Low risk of bias (8/9)

Abbreviations: CDC, Centers for Disease Control and Prevention; NOS, Newcastle–Ottawa Scale.

**Table 5:** Stud ¡es reporting on the relationship between obesity and allergies (*n* = 4)

Author and publication year	Study design	Country	Sample size (*n*)	Age range or mean age	Obesity definition	Outcome definition	Outcome identification	Covariates	Results	Study quality (based on NOS score)
Lei et al.	Cross-sectional	China	3327 (1,663 girls)	2–14 years	Chinese growth charts	Allergic rhinitis and its impact on asthma criteria and atopic dermatitis using the Hanifin and Rajka criteria	Clinical examination by doctor	-	Rhinitis overall: 1.33 (1.04–1.72); girls: 1.48 (1.00–2.18); boys: 1.20 (0.86–1.67). Dermatitis overall: 1.33 (1.02–1.74); girls: 1.42 (0.93–2.16); boys: 1.24 (0.87–1.75)	Low risk of bias (6/8)
Silverberg et al.	Case control	United States	1242 (592 girls)	7 years	WHO growth reference charts for <2 year olds; CDC growth charts for > 2 year olds	International Classification of Diseases–ninth revision diagnostic code 691.8 for atopic dermatitis	Clinical examination by doctor	Sex; season of birth; comorbid asthma, allergic rhinoconjunctivitis and food allergy; race/ethnicity; and immunization up-to-date, age at the time of the study and at first diagnosis of atopic dermatitis, height, height for age, weight, weight for age, head circumference, head circumference for age	Obese status increased odds of atopic dermatitis (OR 2.00; 95% CI 1.22–3.26)	Low risk of bias (8/9)
Skinner et al.	Cross-sectional	United States	2792	3–5 years	CDC growth charts	Clinical guidelines for eczema diagnosis	Parental report of doctor diagnosis of eczema	Age, race/ethnicity, income, insurance status	Obese or very obese status did not increase odds of eczema diagnosis in boys or girls	Low risk of bias (8/8)
Weinmayr et al.	Cross-sectional	Multicentre: Brazil, Estonia, Georgia, Germany, Ghana, Greece, India, Italy, Latvia, Netherlands, New Zealand, Norway, Palestine, Spain, Sweden, Turkey	10 652	9.4 years	Cole et al.	Clinical guidelines for allergic presentations	Clinical examination by trained fieldworker, skin prick test	Sex	Skin prick test = OR 1.13 (0.91; 1.42), examined eczema without wheeze = 2.07 (1.03; 4.17)	Low risk of bias (7/8)

Abbreviations: CDC, Centers for Disease Control and Prevention; NOS, Newcastle–Ottawa Scale.
